# A preliminary study to estimate contact rates between free-roaming domestic dogs using novel miniature cameras

**DOI:** 10.1371/journal.pone.0181859

**Published:** 2017-07-27

**Authors:** Courtenay B. Bombara, Salome Dürr, Gabriel E. Machovsky-Capuska, Peter W. Jones, Michael P. Ward

**Affiliations:** 1 Sydney School of Veterinary Science, The University of Sydney, Camden, Australia; 2 Veterinary Public Health Institute, University of Bern, Liebefeld, Switzerland; 3 The Charles Perkins Centre and School of Life and Environmental Sciences, The University of Sydney, Sydney, Australia; 4 School of Electrical and Information Engineering, The University of Sydney, Sydney, Australia; Atlantic Veterinary College, CANADA

## Abstract

Information on contacts between individuals within a population is crucial to inform disease control strategies, via parameterisation of disease spread models. In this study we investigated the use of dog-borne video cameras–in conjunction with global positioning systems (GPS) loggers–to both characterise dog-to-dog contacts and to estimate contact rates. We customized miniaturised video cameras, enclosed within 3D-printed plastic cases, and attached these to nylon dog collars. Using two 3400 mAh NCR lithium Li-ion batteries, cameras could record a maximum of 22 hr of continuous video footage. Together with a GPS logger, collars were attached to six free roaming domestic dogs (FRDDs) in two remote Indigenous communities in northern Australia. We recorded a total of 97 hr of video footage, ranging from 4.5 to 22 hr (mean 19.1) per dog, and observed a wide range of social behaviours. The majority (69%) of all observed interactions between community dogs involved direct physical contact. Direct contact behaviours included sniffing, licking, mouthing and play fighting. No contacts appeared to be aggressive, however multiple teeth baring incidents were observed during play fights. We identified a total of 153 contacts–equating to 8 to 147 contacts per dog per 24 hr–from the videos of the five dogs with camera data that could be analysed. These contacts were attributed to 42 unique dogs (range 1 to 19 per video) which could be identified (based on colour patterns and markings). Most dog activity was observed in urban (houses and roads) environments, but contacts were more common in bushland and beach environments. A variety of foraging behaviours were observed, included scavenging through rubbish and rolling on dead animal carcasses. Identified food consumed included chicken, raw bones, animal carcasses, rubbish, grass and cheese. For characterising contacts between FRDD, several benefits of analysing videos compared to GPS fixes alone were identified in this study, including visualisation of the nature of the contact between two dogs; and inclusion of a greater number of dogs in the study (which do not need to be wearing video or GPS collars). Some limitations identified included visualisation of contacts only during daylight hours; the camera lens being obscured on occasion by the dog’s mandible or the dog resting on the camera; an insufficiently wide viewing angle (36°); battery life and robustness of the deployments; high costs of the deployment; and analysis of large volumes of often unsteady video footage. This study demonstrates that dog-borne video cameras, are a feasible technology for estimating and characterising contacts between FRDDs. Modifying camera specifications and developing new analytical methods will improve applicability of this technology for monitoring FRDD populations, providing insights into dog-to-dog contacts and therefore how disease might spread within these populations.

## Introduction

Estimation of contact rates between individuals is crucial to inform the spread of disease within populations [1]. Contacts can be categorised as either direct (where physical contact between two animals occurs) or indirect (where no physical contact between two animals occurs), both of which can be effective in transmitting disease (depending on the nature of the disease agent involved). In canines, a range of diseases–such as canine parvovirus and canine distemper virus–can be spread via fomites [2–3]. For others–such as rabies–direct physical contact (principally biting) between individuals is required for transmission [4]. Therefore, a description of the nature of contacts and estimation of contact rates between con- and hetero-specific individuals are needed to describe the spread of diseases within populations, via parameterisation of infectious disease models [1].

In the current study, our focus is on the potential spread of rabies in northern Australia, a region currently free from canine rabies but which is under threat of an incursion due to the eastern spread of the disease in Indonesia [5]. Rabies disease spread pathways in Papua New Guinea have recently been characterised [6] and a rabies disease spread model to inform response strategies in northern Australia has been developed [7]. The northern Australian region is characterised by very low human population densities, mostly in discrete Indigenous communities. Within these communities there are often large dog populations (one dog per five residents, or greater), which are mostly free-roaming [8–10]. Disease transmission, in particular a potential rabies incursion, is likely to be spread via free-roaming domestic dogs (FRDD). For this reason, information of the roaming behaviour of domestic dogs (*Canis familiaris*) and the nature of their intra- and inter-specific contacts with wild dogs and dingoes (*Canis lupus dingo*) that also inhabit this region, is critical for understanding potential disease spread and for planning response strategies.

Bio-logging technologies have made significant contributions to understand how animals utilize their environments and interact among each other [11]. Miniaturize data loggers can collect and store information from multiple sensors–such as global positioning systems (GPS), time depth recorders, accelerometers and temperature thermistors [12]. GPS technology is widely used to observe the roaming behaviour of animals, even over long periods of time [13]. When a substantial proportion of individuals in a population are monitored via GPS and locations are recorded at a sufficiently high frequency, contact rates within the population can be estimated [7]. However, the main disadvantage of only employing GPS loggers is the lack of visual confirmation of the nature of these contacts. Therefore, direct (involving physical interaction) versus indirect contacts (where two dogs are within close proximity but no physical contact occurs) cannot be distinguished. Particularly for contagious diseases driven by direct contact between susceptible and infectious hosts (such as rabies), knowledge and characterisation of the type of contact is essential to provide accurate estimates of potential disease spread.

Cameras have previously been identified as useful tools to collect data on the frequency and characteristics of contacts [14–16]. In a study of deer in Texas, USA [15], deer-borne cameras were used to collect behavioural data from an animal’s perspective. Deer were identified based on distinguishing features and contacts were recorded. A more recent study by Lavelle et al. [16] compared methods of contact estimation using deer-borne detection systems involving a GPS logger, a proximity logger and a video camera on a sample of white-tailed deer. In this study it was reported that contact estimated using GPS data could be an underrepresentation of actual contacts [16]. Recent developments in animal-borne video cameras have provided partial glimpses of fine-scale, detailed behaviours in different animal species, including interactions with their environment [14, 15, 17], social interactions [16, 18, 19], and foraging behaviour [20–22]. This has presented new opportunities for developing the optimal device that would enable researchers to collect their desired data. However, there are also several challenges in implementing this novel technology; these include battery and data storage capacities, weight, packaging and cost-effectiveness (that is, cost of the device and amount of labour required versus the value of the data collected for the specified study goal) [21, 23].

Here, we deployed contact identification systems (a miniaturised video camera combined with a GPS logger) to characterise contacts in FRDD populations in Aboriginal and Torres Strait Islander communities in northern Australia. In addition, we discussed the potential utility of the information generated for disease control via parameterization of models.

## Material and methods

### The deployments: Contact identification system

**Fig 1:**
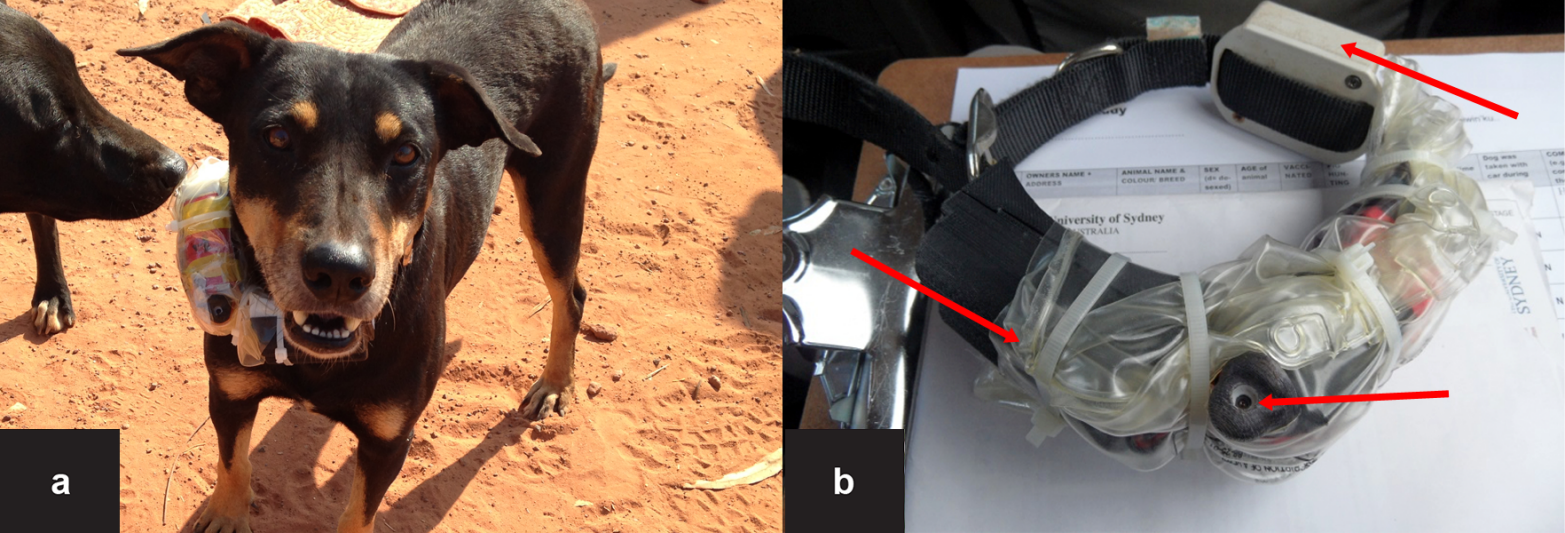
Video-camera collar on a community dog (A) in Galiwin’ku, the Northern Territory, Australia, October 2014. The collar (B) also included a GPS (Global Positioning System; 1) logger in addition to its lens (2) and battery (3).

We customized a miniaturised camera as previously reported [21]. In the present study, the camera (U10 AU USB Flash Drive DRV Camera, DV Taiwan) was enclosed in a 90 x 30 x 20 mm (L x W x H) 3D-printed plastic case and attached to a nylon dog collar specifically secured to reduce collar movement. To protect the camera against sea water and rain, we used an empty saline solution bag (Fig 1A). The camera had a sensor resolution of 720 x 480 HD at 30 frames per second and a 36° lens angle and a storage capability of a MicroSD 64 GB (for more details see [21]). To maximize data collection, the unit was powered by two 3400 mAh Panasonic NCR lithium Li-ion batteries, enabling 20 hr of continuous video recording. A GPS logger (CatLog®; http://mr-lee.com/catlog.htm) which has been used in previous studies in these populations [8–10] was also attached to each collar (Fig 1B). The GPS loggers were set to record locations (latitude and longitude, called GPS fix) every minute. The final contact identification system–including the batteries, camera and GPS logger–weighed 313 g, which is below the 3% threshold (system weight ÷ animal weight) beyond which behavioural disruptions are likely to occur in animals [20].

To assess any potential adverse reaction of the dogs carrying the collars, a dummy camera was constructed and tested on two farm dogs from Kirkham NSW and two farm dogs from Jindabyne NSW, Australia. Our initial trials revealed no negative effects of the use of collars in dogs. The study was approved by the Animal Ethics Committee of The University of Sydney (# N00/7-2013/2/6015).

### Study area and study animals

**Fig 2:**
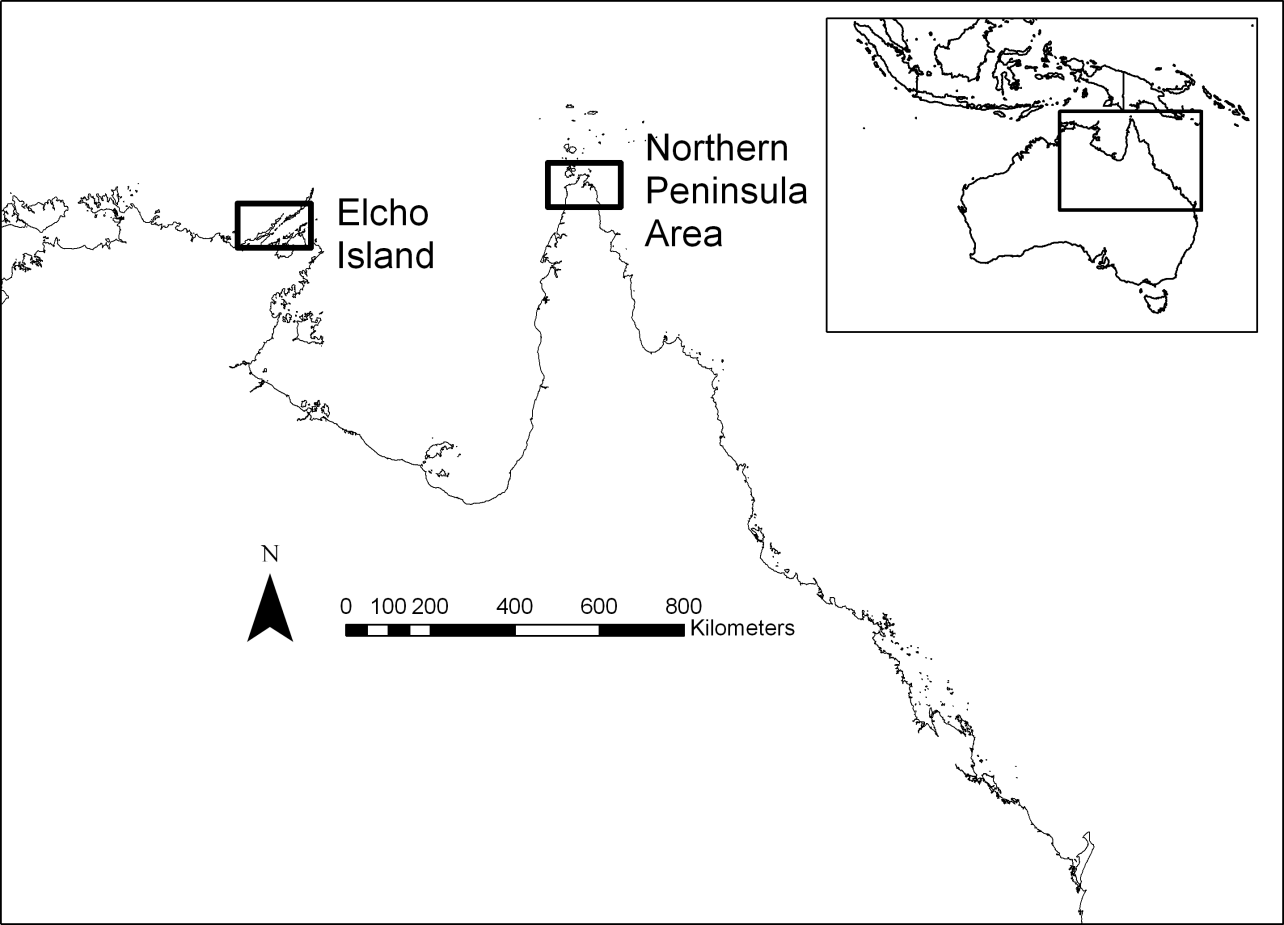
Location of two sites in northern Australia where video-camera collars for visualising and estimating dog-to-dog contacts were trialled on community dogs.

As a pilot study, we deployed these miniaturised cameras and GPS data loggers on six community dogs, two in Seisia in the Northern Peninsula Area (10.883° S, 142.383° E) of Cape York, Queensland, Australia and four in Galiwin’ku on Elcho Island (12.024°S, 135.572°E), Northern Territory, Australia, in September and October 2014, respectively (Table 1 and Fig 2).

**Table 1:** Description of six dogs fitted with video cameras in a study of interactions and contact rates between free-roaming dogs in Indigenous communities in northern Australia. All dogs include were classified as “camp dog” breed.

						Camera on		Camera off	
ID	Sex	Colour	Community	Age	Dog household	Date	Time	Date	Time
42	male, neutered	black, tan & white	Galiwin'ku	adult	single	21/10/14	16:23	22/10/14	11:30
14	male, neutered	black & tan	Galiwin'ku	adult	unknown	22/10/14	15:51	23/10/14	15:16
23	male, entire	brindle	Galiwin'ku	young	multiple	21/10/14	14:00	22/10/14	11:39
115	male, neutered	black & tan	Galiwin'ku	young	multiple	22/10/14	16:06	23/10/14	15:16
130	male, neutered	tan	Seisia, NPA	young	multiple	3/09/14	10:28	4/09/14	10:00
33	male, neutered	tan	Umagico, NPA	adult	multiple	3/09/14	12:15	3/09/14	16:15

Households participating in this study had been selected opportunistically in an earlier study [8]. In conjunction with the local animal management worker, researchers drove around the communities searching for dogs and owners at home who were willing to participate. The study methods were explained to the owners and following verbal consent the dogs were manually restrained and cameras were attached.

### Data analysis

#### Video footage

**Fig 3:**
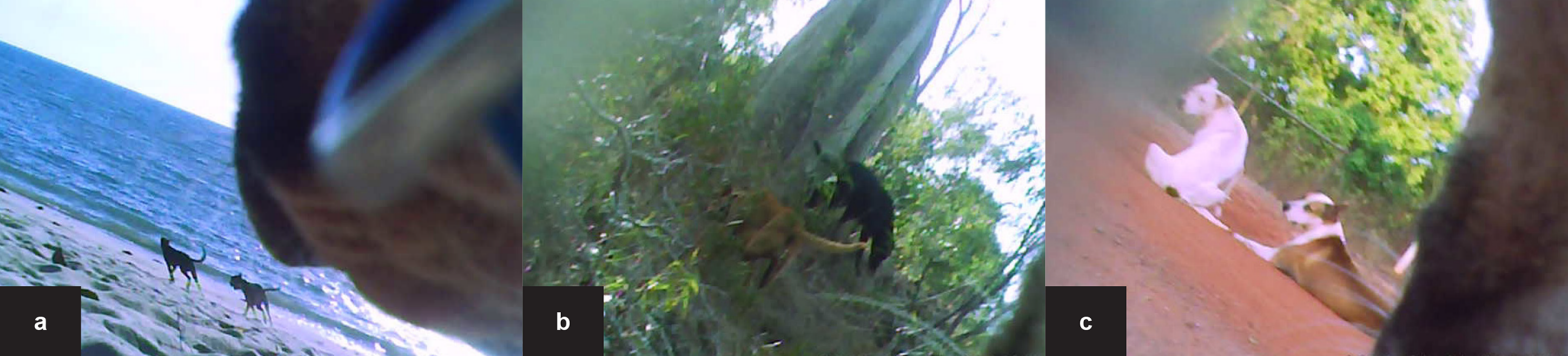
Habitat use identified from dog ID130, with attached video cameras in Seisia, the Northern Peninsula Area (NPA) of Cape York, Queensland. The study was conducted during September 2014. Images include: (a) beach (b) surrounding bush land, (c) urban (road) environment.

Contact rates were analysed (S1 Data) using a dog-to-dog contact definition of being sighted within one five-minute interval of video footage. No spatial limit was set to define a contact via video–as soon as another dog was visible this was counted as a contact. Contacted dogs were identified individually where possible enabling calculation of repeated contacts between the same individuals. The contacts were classified as ‘direct’ if physical contact between two dogs occurred and ‘indirect’ if another dog was visible in the camera field of view but no physical contact occurred. The environment (inside house, surrounding bush, urban environment [houses and other infrastructure visible] and beach; Fig 3) and the time period during which a dog remained in each particular environment were recorded over the entire period of the video and for each contact. The video footage was reviewed using the program Avidemux 2.6.6 (Multi-platform Video Platform Editor, Boston MA). The videos were edited to exclude unusable footage–when the view was obstructed or when it was too dark to observe contacts. The recording of contact data was restricted to daylight hours using the dog-borne video cameras. To estimate daily contact rates to enable comparisons between dogs, the number of contacts were extrapolated to a 24 hr period, assuming constant contact rates during this period.

#### GPS data

GPS fixes were used as a comparative non-visual procedure to calculate contact rates. GPS fixes that were obviously in error or caused by a dog being transported in a vehicle were identified based on the assumption that it is implausible for a community dog to run faster than 20 km/h within a full one-minute period [8]. Thus both GPS fixes at the beginning and end of a one-minute period in which the calculated speed of movement was >20 km/h were excluded from the dataset before further analysis. Contacts between dogs using the GPS loggers can only occur between dogs that both wear a logger. In addition to the six dogs fitted with contact identification systems, six dogs in Seisia and 24 dogs in Galiwin’ku were collared with GPS loggers only [24]. To estimate contacts between dogs the GPS data was searched for concurrent location fixes within the same minute that were less than 20 m apart, based on location error values of the GPS loggers and the one minute interval set for recording GPS fixes [7]. The identification of GPS fixes meeting these conditions was achieved using R (“spacetime” package). To enable a better comparison between the contacts estimated by the video and GPS methods, GPS contacts within the same five minute interval were then counted as one contact.

#### Camera versus GPS contacts comparison

To compare the contact rates estimated by the two deployment tools (camera versus GPS), videos were searched for contacts recorded with other dogs wearing a GPS logger only. Because the video cameras used in this study only produce analysable data during daylight hours, these comparisons were restricted to daylight hours. Therefore dog-borne cameras provided a conservative estimate of contact rates.

**Fig 4:**
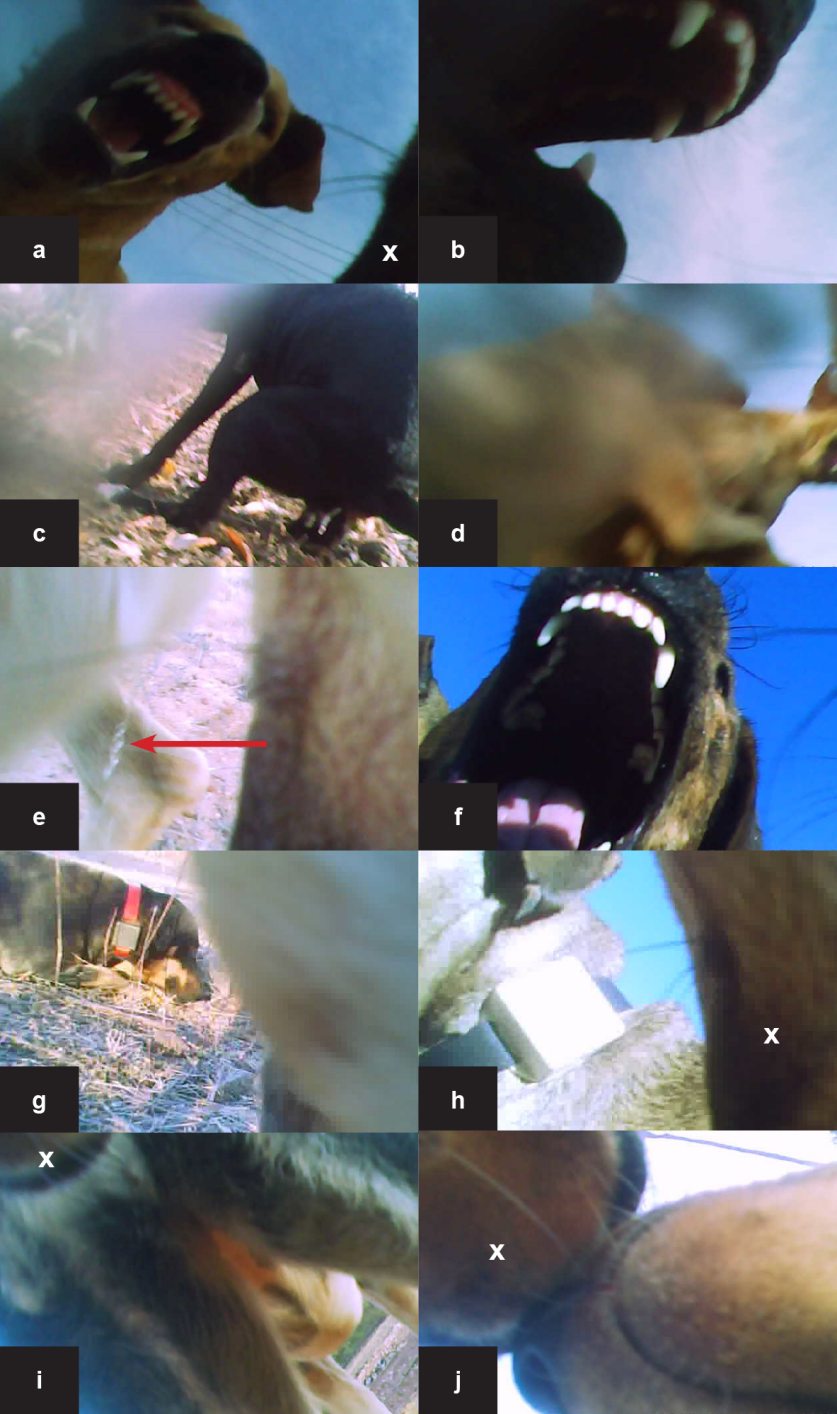
Contact data from cameras attached to community dogs from the Northern Peninsula Area of Cape York, Queensland and Galiwin’ku, East Arnhem Land, the Northern Territory. The study was conducted during September and October 2014. Observations include (a) direct contact during play fight, (b) the same dog moments after (a) indicating direct physical contact during play flight, (c) dog defecating, (d) two dogs play fighting in close contact, (e) female dog urinating [urine indicated by red arrow], (f) direct bite to the camera during a play fight, (g) and (h) GPS loggers from identified dogs included in the GPS study (i) dog sniffing the rear of another dog (j) two dogs touching muzzles. Images (a) to (f) are from dog ID130, images (g) and (h) are from dog ID115. The mandible of the camera-equipped is indicated by the white x where visible.

The GPS devices can only gather contact data between two dogs fitted with GPS collars, however the video camera method is not restricted in this way. To better compare the contact rates estimated by the two deployment tools (camera versus GPS), we isolated contact data derived from the video footage to only include dogs fitted with a GPS device (Fig 4G and 4H). These dogs could be identified based on colouration patterns and visual markings. Contacts recorded by GPS were restricted to the time period when the camera on the respective dog was recording. The contact rates derived from the video camera data analysis are likely to be underestimated because a considerable number of contacts by the same dog could occur within each five minute interval, but were only recorded as a single contact in this study. Therefore, for a better comparison with the GPS method, we also merged multiple GPS contacts within the same five-minute interval into a binary outcome.

## Results

### Video camera data

We recorded a total of 97 hr of video footage from cameras deployed on the six community dogs. Videos were recorded for a duration of 4.5–22 hr (mean = 19.1). The hours of usable footage that was analysed ranged from 2.8–10.8 hr (Table 2). Data from one of the six cameras was excluded from further analysis because the camera was retrieved 4 hr after it was attached and this dog did not move from its initial resting position under a truck. The other cameras were retrieved after 4.5–24 hr without damage.

**Table 2:** Summary of the contact data derived from a video camera collar study of five community dogs and extrapolation of contact data to provide contact estimates over 24 hr in Galiwin’ku, East Arnhem Land, the Northern Territory and the Northern Peninsula Area (NPA) of Queensland, conducted in September and October 2014.

Dog ID	Duration of usable video footage (min)	Contacts				Number of unique^c^ dogs contacted	Estimated total number of contacts per 24 hr	Estimated total number of unique contacts per 24 hr
		Total	Direct^a^		Indirect^b^			
42	185	1		0	1	1	8	8
14	170	11		1	10	3	93	25
23	520	20		13	7	7	55	19
115	560	55		44	11	12	141	31
130	645	66		47	19	19	147	42
Total	2080	153		105	48	42	89^d^	25^d^

^a^where physical contact between two dogs occurred

^b^where dogs were observed in the camera footage from a distance, however no physical contact occurred

^c^dogs which could be individually identified (based on colour patterns and markings)

^d^mean

We observed a wide range of social behaviours (Fig 4). The majority (69%) of all observed interactions between community dogs involved direct physical contact (Table 2). Direct contact behaviours included sniffing, licking, mouthing and play fighting. No contacts appeared to be aggressive however multiple teeth baring incidents were observed during play fights (Fig 4A, 4B, 4D and 4F).

A total of 153 contacts were recorded for the five dogs, equating to daily estimated contact rates of 8, 55, 93, 141 and 147 (Table 2). Using different coloration patterns and visual marks (including those that were fitted with GPS loggers), a total of 42 unique dogs were identified in the videos. The number of unique dogs contacted ranged from 1 to 19 (Table 2). For all five dogs fitted with camera collars, only six contacts were with dogs that could not be identified on coloration patterns and visual marks due to poor visibility.

Video footage provided additional information on habitat utilisation. Most dog activity was observed in urban environments (Table 3), however some dogs were also seen in surrounding bush areas, inside houses and one dog (ID130) was observed occupying the nearby beach (Fig 3A). Dog-to-dog contacts were observed in all habitats occupied by the community dogs (Table 3). Contact rates during periods of monitoring were greater in the bush and at the beach than in an urban environment including inside houses. Interactions between community dogs often occurred early in the morning between 5am and 10am, and contacts were lowest during the middle of the day, between 10am and 3pm (Table 4). One dog (ID23) was confined (not able to freely roam, restricted by fencing or tethering) with a chain at two different times (a total of 2.4 hr) during its 8.7 hr period of monitoring. Few (2/20) contacts were observed while the dog was confined.

**Table 3:** Contact (observation time, minutes) behaviour in habitats occupied by community dogs in Galiwin’ku, East Arnhem Land, the Northern Territory and the Northern Peninsula Area (NPA) of Queensland, conducted in September and October 2014.

	Contacts				
Dog ID	Inside house	Urban	Surrounding bush	Beach	Contacts per hr of observation
42	nil^a^	1 (185)	nil	nil	0.3
14	6 (100)	5 (65)	0 (5)	nil	3.6 / 4.6 / 0
23	nil	20 (520)	nil	nil	2.3
115	nil	30 (475)	25 (85)	nil	3.8 / 17.6
130	nil	42 (555)	17 (60)	7 (30)	4.5 / 17.0 / 14.0

^a^no observation in this environment

**Table 4:** Contact behaviour of community dogs at different times of the day in a video camera study conducted in Galiwin’ku, East Arnhem Land, the Northern Territory and the Northern Peninsula Area (NPA) of Queensland, September and October 2014.

Dog ID	Time of day	Contacts	Duration of usable video footage (min)	Contacts per hr observation
42	5am–10am	0	55	0
	10am–3pm	0	0	0
	3pm–8pm	1	130	0.5
	Total	1	185	**0.3**
14	5am–10am	0	0	0
	10am–3pm	0	0	0
	3pm–8pm	11	170	3.9
	Total	11	170	**3.9**
23	5am–10am	17	225	4.5
	10am–3pm	0	20	0
	3pm–8pm	3	275	0.7
	Total	20	520	**2.3**
115	5am–10am	22	240	5.5
	10am–3pm	27	185	8.8
	3pm–8pm	6	135	2.7
	Total	55	560	**5.9**
130	5am–10am	30	135	13.3
	10am–3pm	6	270	1.3
	3pm–8pm	30	240	7.5
	Total	66	645	**6.1**

### GPS data

The GPS loggers recorded 933 (ID130), 416 (ID14) and 624 (ID42) fixes for three dogs during the time period of video recording, after having excluded 25, 4 and 0 GPS fixes, respectively, based on the exclusion criteria of > 20 km/h. However, the GPS loggers failed to record fixes for the remaining three dogs. The total numbers of contacts with other dogs in the same community estimated from the GPS data during that period were 3, 1 and 16 for dogs ID130, 14 and 42, respectively (Table 5).

**Table 5:** Contacts between paired GPS-collared community dogs in East Arnhem Land, the Northern Territory and the Northern Peninsula Area (NPA) of Queensland, in September and October 2014. Contacts are calculated when concurrent GPS fixes are within 20 m and 1 min.

		GPS			Video		
Dog 1	Dog 2	Estimated contacts during day/night time^b^	Days in common^c^	Contacts per day	Estimated contacts	Days in common^d^	Contacts per day
Seisia_130	Seisia_11	3 / 0	0.88	3.41	5	0.91	5.50
Seisia_130	Seisia_12^a^	**–**	**–**	**–**	5	0.91	5.50
Seisia_130	Seisia_28	0 / 0	0.56	0	2^e^	0.91	2.20
Seisia_130	Seisia_124^a^	**–**	**–**	**–**	17	0.91	18.68
Seisia_130	Seisia_123^a^	**–**	**–**	**–**	2	0.91	2.20
	**TOTAL**	**3 / 0**	**0.72**	**4.17**	**31**	0.91	**34.08**
Galiwinku_14	Galiwinku_17	1 / 0	0.48	2.08	0	0.54	0
	**TOTAL**	**1 / 0**	**0.48**	**2.08**	**0**	**0.54**	**0**
Galiwinku_42	Galiwinku_07	1 / 2	0.79	3.80	0	0.60	0
Galiwinku_42	Galiwinku_101	2/ 11	0.79	16.46	0	0.60	0
	**TOTAL**	**16**	**0.79**	**20.25**	**0**	**0.60**	**0**

^a^GPS logger failed

^b^day time was defined between sunrise (06:03) and sunset (18:24) at 15^th^ of October 2014 in Galiwin’ku, according to the Geoscience Australia website (http://www.ga.gov.au/bin/geodesy/run/sunrisenset)

^c^number of days during which both dog 1 and dog 2 were wearing a GPS collar simultaneously

^d^number of days during which the camera on dog 1 was recording and the GPS collar was attached to dog 2

^e^one of the two contacts occurred outside the recording time of the GPS unit

### Camera versus GPS contacts comparison

Footage from two (ID115 and ID130) of the five dogs equipped with video cameras showed contact with dogs equipped with GPS loggers. Dog ID115 contacted ID120 on two occasions at (24 hr time) 18:23 and 18:26. Dog ID130 was observed to come into contact with five other GPS collared dogs (ID11, 12, 28, 124 and 123) on 31 occasions during the video camera monitoring period (Table 5). A contact rate of 34 per 24 hr was estimated using the video camera between GPS collared dogs. GPS loggers on dog ID12, 123 and 124 malfunctioned and therefore did not record any data. While two video contacts between dog ID130 and dog ID28 were observed, only one of these contacts occurred while the GPS device was recording data; based on the GPS data, a contact between those two dogs was not recorded. Five and three contacts were recorded between dog ID130 and dog ID11 using the video camera footage and the GPS data, respectively. These contacts occurred during similar time periods (16:56–17:05 for the GPS contacts and 16:58, 17:03, 17:09, 17:14 and 17:19 for the video observed contacts). Dog ID 14 and 42 recorded contacts via GPS logger only, of which one (06:37) and three (17:24, 17:56, 18:16) occurred during day time (before sunset) and 13 after sunset (20:54–04:21), when the video camera was not able to record visible footage (Table 5).

## Discussion

This study demonstrates the value of video camera collars for descriptive characterisation of contacts and foraging behaviours of FRDDs in remote Indigenous communities. It also demonstrates that both video camera collars and GPS loggers can be used to estimate contact rates between FRDDs. Disease transmission is a complex process and comparisons of technologies to monitor contact data can provide a more complete understanding of disease dynamics [16]. Moreover, transmission parameters informed by contact rates are a crucial step in modelling disease spread and devising appropriate control strategies [5].

The free-roaming nature of dogs has consistently been recognised as a risk factor for disease transmission [25, 26]. However, the mechanisms of disease transmission are difficult to visualise in such populations. For characterising contacts between FRDDs, several benefits of analysing videos compared to GPS fixes alone were identified in this study. Most importantly, videos provide information on the nature of the contact between two dogs, a crucial determinant of disease transmission. Some diseases may require direct, physical contact (e.g. bites for rabies or close contacts for canine distemper, see Fig 4B and 4J as an example) for transmission. For other diseases larger distances between the individuals might be sufficient, or transmission might be via indirect contacts through contaminated environments or fomites (e.g. canine parvovirus that can be spread via contact with canine faeces, see Fig 4C and 4I as an example). Dogs were observed contacting faeces and urine from other dogs (see Fig 4C and 4E as an example). Also, not only information on dogs enrolled in the study was collected, but dogs with video cameras can be used (as “sentinels”) to record contacts between other dogs as well (see Fig 4D as an example).

Rabies infection influences animal behavior, presenting either as the furious or paralytic forms, and will thus change the characteristics of observed contacts. Although a lack of aggressive interactions were identified in the present, video camera collars would be particularly useful for documenting effective contacts for rabies transmission, because dog bites and teeth baring instances were easily observed on the video footage (and these contacts were often close up and front on).

A simplifying assumption often made in disease spread models is homogenous mixing of individuals within a population, with constant contact rates over time and within different environments. However, such an assumption is seldom realistic. Aggregations of dogs are expected to promote disease spread, depending on when and where such non-homogenous mixing occurs. Majumder et al. [27] found that social aggregates of dogs tended to be most common during foraging forays, away from their households in a population of FRDDs in urban India. This finding was consistent with our study in which contacts were fewer in houses and surrounding urban environments than further away from the dogs’ homes, such as the surrounding bush and the beach. Also, it was evident from the video footage in the present study that confining dogs by tethering considerably restricts the number of contacts: dog ID23 had 2.8-times more contacts when it was free-roaming as when it was confined. Restricting the movement of FRDDs is likely a useful control point to prevent disease transmission [5, 7] and is an action that community members can implement in the case of a disease outbreak [28].

**Fig 5:**
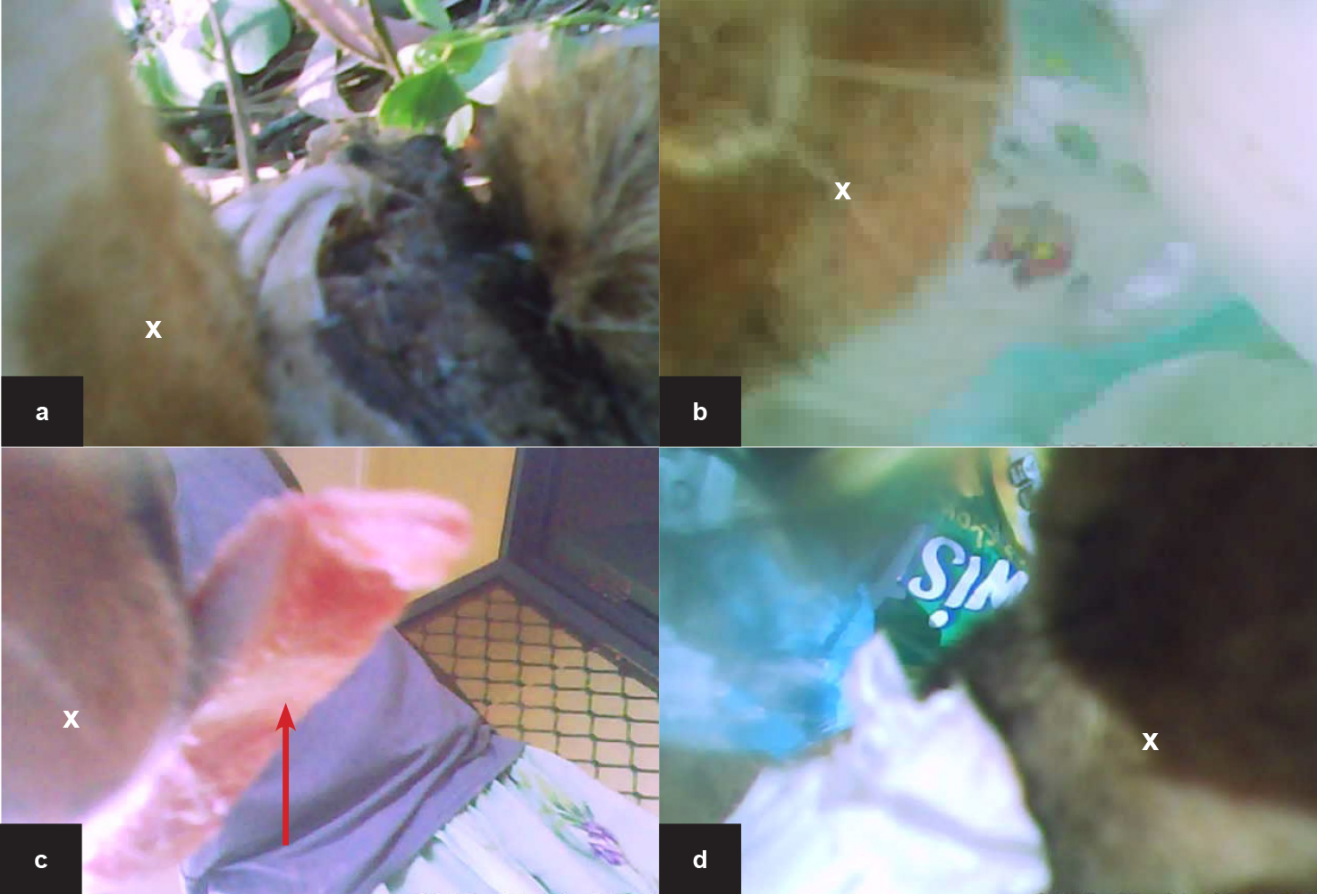
Foraging data captured with video camera collar on community dogs, in Galiwin’ku, the Northern Territory. The study was conducted during September and October 2014. Images include: (a) dead animal, canine tooth indicated by red arrow, (b) nappy, (c) dog eating raw bones indicated by red arrow, (d) rubbish. The mandible of the camera-equipped dog in each image is indicated by the white x, images (a) to (c) are from dog ID115, image (d) is from dog ID130.

Video footage also provides qualitative information regarding the circumstances in which contacts might occur. A number of feeding and foraging behaviours were observed in video camera footage, some of which are shown in Fig 5. Foraging behaviours observed included scavenging through rubbish and rolling on dead animal carcasses. Identified food consumed included chicken, raw bones, animal carcasses, rubbish, grass and cheese. Many of the foraging behaviours observed have implications for disease transmission. For example, one dog (ID115) was observed eating a nappy (diaper). This is consistent with the findings of Brown (2006) who reported that 35% of dog faecal samples from Indigenous communities contained nappy remnants. Coprophagy of human faeces and scavenging may facilitate the lifecycles of potentially zoonotic pathogens [29–31] and may contribute to physical transmission of non-zoonotic human disease e.g. if a dog licks a child after eating a nappy [32].

**Fig 6:**
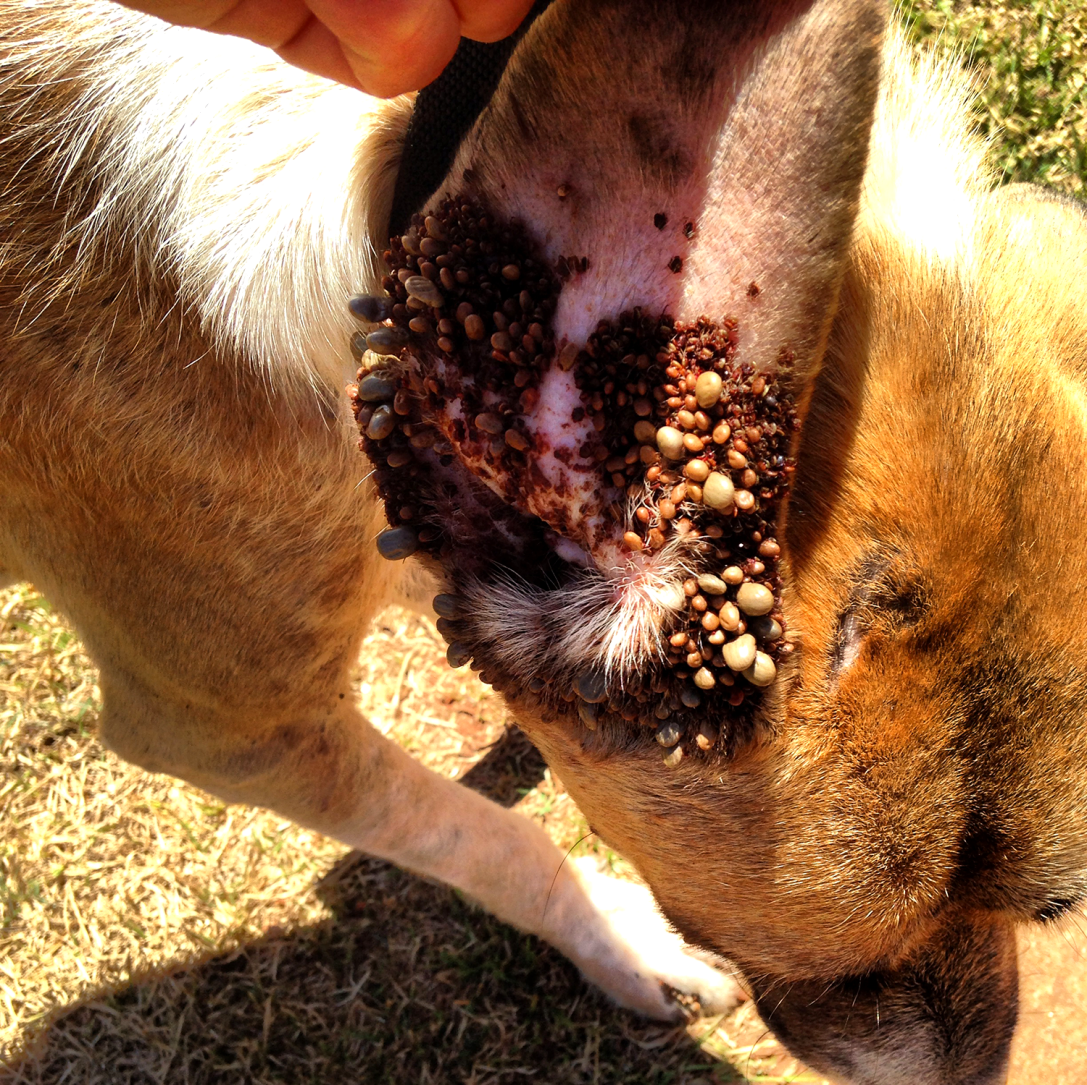
Tick infestation on the pinna of a community dog in Umagico, the Northern Peninsula Area (NPA) of Cape York, Queensland. September 2014. Photo credit: C. Bombara.

Contact with dog faeces was also observed (ID130), which is a major transmission point for certain diseases such as canine parvovirus [2]. Dog faeces within communities also increase the risk of zoonotic transmission of *Echinococcus granulosus* to humans [33]. In addition, consumption of raw meat and dead animal carcasses were observed in the current study; both of these behaviours have been recognized as risk factors for *E*. *granulosus* infection in dogs [33–34]. Furthermore, dogs were observed to spend a considerable amount of time in the bush where–based on field observations–contact with ticks is likely (Fig 6). This has been recognised as a major risk factor for canine vector-borne diseases identified in Indigenous community dogs [25, 35]. Four of the five dogs fitted with cameras were observed to be fed outside the house by a human on one or more occasions. Such information about feeding, foraging behaviour and nutrition might also be useful when designing disease control programs, especially if these involve restricting the movement of FRDDs.

Cameras have the potential to validate accuracy of GPS data and *vice versa*, since the position (location and time) of dogs wearing GPS collars can be identified in the recorded videos (see Fig 4G and 4H as examples). The two different methods used in this study to investigate contact rates–the video camera collars and GPS loggers–differ in many aspects, which also caused differences in the estimated contact rates. For dog ID130, 4.17 contacts per day were recorded using the GPS loggers, whereas the contacts between the same dogs (ID11 and ID28) using the video technique resulted in 7.7 contacts per day (7 contacts during a 0.91 day period). For other dogs (ID14 and ID42), contacts were only recorded by the GPS loggers and were not seen in the video. There are many reasons for these differences. First, contacts were defined using different spatiotemporal thresholds for each of the technologies. This highlights the influence of user-specified definitions on contact estimations. In the case of analysis of the GPS data, the calculated contact rates were particularly sensitive to the GPS spatiotemporal thresholds used to define a contact (within 20 m within the same minute). In contrast, when analysing video data a contact was defined as sighting another dog (without a specific spatial threshold being set) within one five-minute interval. The contact rates are likely to be underestimated because a considerable number of contacts by the same dog could occur within each five-minute interval. Therefore, we also defined several contacts recorded by the GPS within the same 5 minutes period as one single contact to better compare the two methods.

Both devices were unable to record contacts at some times. GPS-based contacts are estimated only between dogs with fitted GPS loggers, whereas the cameras record contacts with all dogs within the lens field of view. The video camera has a limited field of view facing forward, whereas contacts are estimated within a predefined radius of each dog fitted with a GPS logger. For example, for video cameras no contacts could be recorded when the camera lens was obscured (including very close contacts, when the dog was laying on the lens or obstruction by the dog’s mandible), and for GPS loggers fixes could not be recorded when satellite interference occurred. The restriction of video camera collars to daylight hours further limited the time frame in which contacts were recorded for video cameras. For example, the daily video camera contacts for dog ID130 were estimated from a small proportion of daily activity (10.8 hr out of the total recorded video time of 22 hr). We extrapolated the number of contacts from a small amount of video footage to estimate daily contact rates, enabling comparisons between dogs. We acknowledge that these rates might vary considerably from actual contacts because we were unable to observe activity of the dogs during the night time periods. Our preliminary findings indicate that, beside the high heterogeneity between individuals, more contacts occur in the evenings and mornings than in the middle of the day. Because we were unable to use video footage captured during night time periods, we might have underestimated contacts via this method. However, this provides a baseline estimate as a comparison in future studies. Improvement in the video camera technique would therefore include an infrared camera (but requiring additional battery power) and a wide-angle lens.

Regardless of the technology used, inaccuracy in contact estimates needs to be considered when using such information to inform disease spread models. A sufficient number of FRDDs needs to be monitored in a population on more than one occasion to generate robust contact rate estimates. Nevertheless, we demonstrated that the two devices provided useful contact data and complementary information, e.g. cameras provided qualitative (characteristics of contacts) and quantitative contact data however GPS loggers are a more feasible means of gathering quantitative contact data for a larger population of dogs in the field due to greater cost-effectiveness. Qualitative, visual representations of dog behaviour are invaluable when assessing effective contacts between dogs.

Additional challenges for the video camera devices include battery life, robustness and the laborious analysis of the video footages. Possibilities to increase battery life include a lower power processing chip or motion triggering video cameras, as used by Lavelle et al. [16]. However, at our study sites with high frequencies of contacts and movements, motion triggered videos would be expected to activate often (more than in wildlife studies) and thus might not provide a solution. Robustness is crucial: damage can be caused by a range of factors such as water (in rivers and sea or heavy rain during the monsoon season), fights between dogs or unwarranted handling of the collar by people. Although no substantial problems were observed in our study and no cameras were damaged, increased robustness is desirable bearing in mind the weight of the camera collar and practical limitations for attachment. Finally, the extraction of valuable data requires the examination of a large amount of extremely unsteady video footage. To reduce unsteady footage a camera stabiliser (e.g. Steadicam) could be incorporated) [36]. Development of semi-automated methods using machine-learning techniques will accelerate the analysis of footage increasing the practicality and viability of large-scale use of dog-borne video cameras in epidemiological studies.

## Conclusions

This study demonstrates that dog-borne video cameras are a valuable technology for characterising contacts between FRDDs. Our study demonstrates that there are considerable variations in contacts between dogs and at different times of the day, therefore extrapolation of contact data to a wider population of dogs and to a longer time period should be done with care. Modifying camera specifications and developing methods for the analysis of large volumes of often unsteady footage remain challenges to be overcome before such systems can be regularly deployed in FRDD populations to monitor dog-to-dog contacts and before they could be considered as an easily applicable tool to inform epidemic models of disease transmission. However, dog-borne video cameras provide invaluable evidence of dog behaviour in the field that might influence disease transmission.

## Supporting information

S1 Data GPS data collected and included in analysis of contact rates for two in Seisia in the Northern Peninsula Area (10.883° S, 142.383° E) of Cape York, Queensland, Australia and four in Galiwin’ku on Elcho Island (12.024°S, 135.572°E), Northern Territory, Australia, in September and October 2014, respectively. (ZIP)
